# Clinicopathologic characteristics and predictive nomogram of skip lymph node metastasis in gastric cancer

**DOI:** 10.3389/fonc.2025.1590133

**Published:** 2025-08-12

**Authors:** Xinwei Zhang, Yuwei Du, Yuegang Li, Chi Xue, Zhi Zhu

**Affiliations:** Department of Surgical Oncology, The First Affiliated Hospital of China Medical University, Shenyang, China

**Keywords:** gastric cancer, skip metastasis, nomogram, clinicopathological features, prognosis

## Abstract

**Background:**

Lymph node (LN) status is an independent factor affecting the prognosis of gastric cancer patients. Skip metastasis is a pattern of LN metastasis across a peritumoral (PT) area to an extra-peritumoral (EP) area. The purpose of the present study was to determine the clinical characteristics and establish a predictive nomogram for skip metastasis in gastric cancer.

**Methods:**

We reviewed the records of 1657 gastric cancer patients at the First Affiliated Hospital of China Medical University. The patients were categorized in four groups: only station II (skip group); only station I (PT-only group); station I and station II (PT+EP group); and no metastatic LNs (N0 group). The clinical characteristics between the skip group and the other three groups were compared.

**Results:**

The incidence of skip metastasis was 3.3% (55/1657) among the gastric cancer patients. The most common locations of skip metastasis were the No. 7 (50.9%), No. 8a (32.7%), No. 9 (21.8%), and No. 1 groups (20.0%). The skip group had significant differences compared to the PT+EP group in pN stage, Borrmann type, pT stage and location, as predictors in the nomogram for prediction of skip metastasis. The area under the ROC curve was 0.908.

**Conclusions:**

This study identified key risk factors for skip lymph node metastasis and developed the first clinically applicable predictive model. The resulting nomogram demonstrated high accuracy in risk stratification, providing a visual tool to optimize lymph node dissection strategies. These findings support incorporating metastatic location into gastric cancer staging systems and the location of LN metastasis should be considered to improve the LN staging.

## 1 Introduction

As one of the most common malignancies worldwide, gastric cancer is the second leading cause of malignancy-related deaths (1–4). Although the incidence and mortality of gastric cancer have decreased, gastric cancer is still a major threat to public health. Some studies have indicated that lymph node (LN) status is the most important prognostic factor in gastric cancer (5–7) and is an appropriate guide for treatment (8–10), especially involving endoscopic mucosal resection or endoscopic submucosal dissection (11, 12). LN metastasis usually spreads in relation to the lymphatic stream. Metastasis that appears to pass over the peritumoral (PT) area to the extra-peritumoral (EP) area is referred to as skip metastasis. Skip metastasis has been reported in a number of cancer types, including breast, colon, and lung cancers (9, 13, 14). The mechanism underlying skip metastasis in gastric cancer has not been established (15).

In the current study we reviewed our database and the previous literature to establish a predicted nomogram for the risk of skip metastasis in gastric cancer. Nomograms have been developed to quantify risk factors for LN metastasis in other carcinomas (16, 17), but the nomogram developed herein is the first for skip metastasis in gastric cancer. This nomogram can assist in determining the prognosis and treatment strategy in patients with gastric cancer (15, 18).

## 2 Methods

### 2.1 Included participants

This study involved 1657 gastric cancer patients who were treated in the Department of Surgical Oncology at the First Affiliated Hospital of China Medical University in Shenyang, China, between January 1998 and August 2012. The inclusion criteria were as follows: (1) patients who underwent surgery and achieved a radical (R0) resection; and (2) the pathologic information was complete. The exclusion criteria were as follows: (1) patients with distant metastasis, including liver and peritoneal metastases; and (2) patients who underwent neoadjuvant chemotherapy or had a history of malignant tumors.

Patient characteristics, including gender, age, tumor size, location, pT stage, pN stage, Borrmann type, Lauren type, histologic findings, gastrectomy type, lymphovascular invasion, and number of retrieved lymph nodes (RLNs), were obtained from medical records. All of the data were prospectively collected and retrospectively reviewed. The survival of each patient was verified on the basis of hospital records, telephone calls, and the database of the Department of Surgical Oncology. This study was approved by the First Affiliated Hospital of China Medical University.

### 2.2 Definitions of the skip group and other groups

The LNs around gastric cancer were categorized in terms of PT area (station I) and EP area (station II) in accordance with the rules of the Japanese Gastric Cancer Association (JGCA). The 1657 gastric cancer patients were categorized as follows: (1) skip group (n = 55), metastatic LNs distributed only in station II; (2) PT-only group (n = 411), metastatic LNs distributed only in station I; (3) PT+EP group (n = 391), metastatic LNs localized to stations I and II; and (4) N0 group (n = 800), no metastatic LNs.

### 2.3 statistical analysis and nomogram

All statistical analyses and graphics were performed using the SPSS 20.0 statistical package (SPSS Inc., Chicago, IL, USA) and R version 2.11.1 (The R Foundation for Statistical Computing, Vienna, Austria). Categorical variables were compared using the Pearson’s chi-square test, and continuous variables were compared using the Mann–Whitney U test. The survival data of each group was presented as the 5-year disease-free survival (DFS), which was defined as recurrence or death from any cause postoperatively, and the Kaplan–Meier method was used to compare the prognosis in LN metastasis (pN) and LN metastasis rate (rN) staging. LN metastasis (pN) staging is based on the number of LN metastases, while the LN metastasis rate (rN) staging is based on the ratio of the number of positive LNs-to-the total number of RLNs. Risk factors for LN metastasis were studied using a binary logistic regression modeling technique. A P value < 0.05 was considered statistically significant.

A nomogram was developed to identify patients at risk for LN metastasis. The nomogram provides a graphical representation of the factors that can be used to calculate the risk of LN metastasis for an individual patient by the points associated with each risk factor. Nomograms fulfill our desire for visualization of risk probability and allow for personalized treatment (19). The discriminatory ability of the nomogram was represented by receiver operating characteristic (ROC) curve analysis and area under the curve (AUC) values.

## 3 Results

### 3.1 Incidence of skip metastasis

A total of 1657 gastric cancer patients who underwent curative gastrectomy met the inclusion criteria (**Table 1**). Among the final cohort of patients enrolled in the present study, 857 of 1657 (51.7%) had metastatic LNs, 411 of 857 (48.0%) had metastatic LNs around the tumor only, and 391 of 857 (45.6%) had metastatic LNs in the PT and EP areas. The incidence of skip metastasis was 6.4% (55 of 857) among the patients with metastatic LNs and 3.3% (55 of 1657) among the overall gastric cancer population.

**Table 1:** Baseline characteristics of the overall patient population.

Characteristics n(%)		Skip(n=55)	PT-only (n=411)	p	PT+EP (n=391)	p	N0(n=800)	p
**Gender**				0.740		0.443		0.874
	Male	40(72.7)	310(75.4)		261(66.8)		592(74.0)	
	Female	15(27.3)	101(24.6)		130(33.2)		208(26.0)	
**Age (years)**				0.924		0.247		0.289
	≤60	30(54.5)	227(55.2)		245(62.7)		494(61.8)	
	>60	25(45.5)	184(44.8)		146(37.3)		306(38.3)	
**Tumor size (cm)**				0.676		**0.041**		**0.002**
	≤4	26(47.3)	182(44.3)		130(33.2)		539(67.4)	
	>4	29(52.7)	229(55.7)		261(66.8)		261(32.6)	
**Location**				0.120		**0.041**		0.619
	Lower	44(80.0)	265(64.5)		249(64.7)		571(71.4)	
	Middle	5(9.1)	56(13.6)		80(19.4)		117(14.6)	
	Upper	6(10.9)	71(17.3)		43(11.0)		100(12.5)	
	≥2/3 stomach	0(0.0)	19(4.6)		19(4.9)		12(1.5)	
**pT stage**				0.493		0.181		**<0.001**
	T1	6(9.1)	31(7.5)		14(3.6)		344(43.0)	
	T2	5(10.9)	86(20.9)		58(14.8)		155(19.4)	
	T3	26(47.3)	166(40.4)		152(38.9)		201(25.1)	
	T4	18(32.7)	128(31.1)		167(42.7)		100(12.5)	
**pN stage**				0.058		**<0.001**		--
	N0	--	--		--		800(100.0)	
	N1	37(67.3)	221(53.8)		30(7.7)		–	
	N2	13(23.6)	134(32.6)		129(33.0)		–	
	N3a	5(9.1)	46(11.2)		162(41.4)		–	
	N3b	0(0.0)	10(2.4)		70(17.9)		–	
**Borrmann type**				0.141		**0.001**		**<0.001**
	Borrmann I	9(16.4)	48(11.7)		18(4.6)		360(45.0)	
	Borrmann II	5(9.1)	52(12.7)		60(15.3)		132(16.5)	
	Borrmann III	41(74.5)	285(69.3)		282(72.1)		288(36.0)	
	Borrmann IV	0(0.0)	26(6.3)		31(7.9)		20(2.5)	
**Lauren type**				0.306		0.769		**0.023**
	Intestinal	25(45.5)	217(52.8)		186(47.6)		488(61.0)	
	Diffuse	30(54.5)	194(47.2)		205(52.4)		312(39.0)	
**Histology**				**0.015**		**0.011**		**0.049**
	Middle&High	25(45.5)	120(29.2)		112(28.6)		260(32.5)	
	Low	30(54.5)	291(70.8)		279(71.4)		540(67.5)	
**Gastrectomy type**				0.562		0.060		0.479
	Subtotal	48(87.3)	344(83.7)		294(75.2)		723(90.4)	
	Total	7(12.7)	67(16.3)		97(24.8)		77(9.6)	
**Lymphovascular invasion**				0.858		0.876		0.308
	NO	43(78.2)	328(79.8)		302(77.2)		668(83.5)	
	YES	12(21.8)	83(20.2)		89(22.8)		132(16.5)	
**RLNs**				0.123		0.698		**0.013**
	<15	10(18.2)	115(28.0)		63(16.1)		276(34.5)	
	≥15	45(81.8)	296(72.0)		328(83.9)		524(65.5)	

Significant p values are in bold.

*PT* peritumoral, *EP* extraperitumoral, *RLNs* retrieved lymph nodes.

The profiles of the 55 patients with skip LN metastases are shown in **Table 2**. The most common locations of skip metastasis were the No. 7 (50.9%), No. 8a (32.7%), No. 9 (21.8%), and No. 1 groups (20.0%).

**Table 2:** The location of skip metastasis and the number of involved LNs.

Location of skip LNs n(%)	Number of patients (n=55)	Number of metastatic LNs
**Group 1**	11(20.0%)	11
**Group 2**	4(7.3%)	4
**Group 6**	1(1.8%)	3
**Group 7**	28(50.9%)	67
**Group 8a**	18(32.7%)	30
**Group 9**	12(21.8%)	17
**Group 10**	2(3.6%)	2
**Group 11**	1(1.8%)	2
**Group 12**	3(5.5%)	4

*LNs* lymph nodes.

### 3.2 Baseline characteristics of each group

The baseline characteristics of the patients with skip metastasis were compared with the PT-only, PT+EP, and N0 groups, as shown in **Table 1**.

The age, gender, gastrectomy type, and lymphovascular invasion of the skip group were similar to the PT-only, PT+EP, and N0 groups. There was no significant difference between the skip group and the PT-only group, except for histologic findings. The differentiation of the skip group was higher than that of the PT-only, PT+EP, and N0 groups (*p* = 0.015, *p* = 0.011 and *p* = 0.049, respectively). Borrmann III type was more frequent in the skip group than the PT+EP and N0 groups (*p* = 0.001 and *p* < 0.001, respectively). In addition, skip metastasis patients were more likely to have smaller tumors, a lower location in the stomach, and a lower pN stage compared to the PT+EP group. The skip metastasis group tended to have diffuse type tumors, larger tumors, more advanced pT stage, and a greater number of RLNs compared to the N0 group.

### 3.3 Nomogram for prediction of metastatic LNs

Because there was no significant difference between the skip group and the PT-only group, a nomogram for predicting skip metastasis risk was not supported (**Table 1**). Based on multivariable logistic regression analysis (**Tables 3**, **4**), the skip metastasis group tended to have a lower pN stage (*p* < 0.001) and Borrmann type (*p* = 0.037) compared with the PT-EP group, while the skip metastasis group had a more advanced pT stage (*p* < 0.001), higher differentiation (*p* = 0.008), and a greater number of RLNs (*p* = 0.010) compared to the N0 group. Furthermore, we observed that the pT stage and tumor location were highly correlated with independent factors (pN stage and Borrmann type; *p <*0.05; **Table 5**). Predictors of skip metastasis included pN stage, Borrmann type, pT stage, and location in the nomogram based on the skip metastasis group versus the PT+EP group. The predictive factors based on the skip metastasis group versus N0 group (**Table 4**) include pT stage, histologic findings, and number of RLNs. The predicted risk range for skip metastasis > 0.5 cannot be applied in practice.

**Table 3:** Multivariable logistic regression for prediction of skip metastasis (skip vs PT+EP).

Characteristics		*OR*	*p*
**Tumor size (cm)**			0.702
	≤4	1.000	
	>4	1.157(0.549-2.440)	
**Location**			0.239
	Lower	1.000	
	Middle	0.301(0.095-0.946)	0.040
	Upper	0.870(0.290-2.607)	0.804
	≥2/3 stomach	0.000	0.998
**pN stage**			**0.000**
	N1	1.000	
	N2	0.077(0.034-0.174)	0.000
	N3a	0.023(0.008-0.066)	0.000
	N3b	0.000	0.996
**Borrmann**			**0.037**
	Borrmann I	1.000	
	Borrmann II	0.114(0.025-0.519)	0.005
	Borrmann III	0.519(0.172-1.568)	0.245
	Borrmann IV	0.000	0.998
**Histology**			0.117
	Middle&High	1.000	
	Low	0.546(0.257-1.163)	

Significant p values are in bold.

*OR* odds ratio, *PT* peritumoral, *EP* extraperitumoral.

**Table 4:** Multivariable logistic regression for prediction of skip metastasis (skip vs N0).

Characteristics		*OR*	*p*
**Tumor size (cm)**			0.937
	≤4	1.000	
	>4	1.026(0.546-1.926)	
**pT stage**			**0.000**
	T1	1.000	
	T2	7.414(1.457-37.726)	0.016
	T3	23.550(5.019-110.503)	0.000
	T4	33.162(6.727-163.477)	0.000
**Borrmann**			0.075
	Borrmann I	1.000	
	Borrmann II	0.156(0.036-0.684)	0.014
	Borrmann III	0.460(0.135-1.570)	0.215
	Borrmann IV	0.000	0.998
**Lauren type**			0.027
	Intestinal	1.000	
	Diffuse	1.985(1.083-3.641)	
**Histology**			**0.008**
	Middle&High	1.000	
	Low	0.436(0.236-0.804)	
**RLNs**			**0.010**
	<15	1.000	
	≥15	2.647(1.268-5.527)	

Significant p values are in bold.

*OR* odds ratio, *PT* peritumoral, *EP* extraperitumoral, *RLNs* retrieved lymph nodes.

**Table 5:** Relationship between pN stage and Borrmann type with pT stage and location (skip vs PT+EP).

pT stage and location (n)	pN stage				*p*	Borrmann type				*p*
	N1(%)	N2(%)	N3a(%)	N3b(%)		Borrmann I (%)	Borrmann II(%)	Borrmann III(%)	Borrmann IV(%)	
pT stage					**0.001**					**<0.001**
T1	6(31.6%)	10(52.6%)	3(15.8%)	0(0.0%)		19(100.0%)	0(0.0%)	0(0.0%)	0(0.0%)	
T2	16(25.0%)	18(28.1%)	25(39.1%)	5 (7.8%)		0(0.0%)	16(25.0%)	44(68.8%)	4(6.3%)	
T3	30(16.9%)	57(32.0%)	66(37.1%)	25(14.0%)		3(1.7%)	29(16.3%)	140(78.7%)	6(3.4%)	
T4	15(8.1%)	57(30.8%)	73(39.5%)	40(21.6%)		5(2.7%)	20(10.8%)	139(75.1%)	21(11.4%)	
Location					0.454					**<0.001**
Lower	47(16.0%)	89(30.4%)	115(39.2%)	42(14.3%)		21(7.2%)	45(15.4%)	210(71.7%)	17(5.8%)	
Middle	11(12.9%)	25(29.4%)	33(38.8%)	16(18.8%)		3(3.5%)	8(9.4%)	70(82.4%)	4(4.7%)	
Upper	8(16.3%)	19(38.8%)	15(30.6%)	7(14.3%)		2(4.1%)	9(18.4%)	36(73.5%)	2(4.1%)	
≥2/3 stomach	1(5.3%)	9(47.4%)	4(21.1%)	5(26.3%)		1(5.3%)	3(15.8%)	7(36.8%)	8(42.1%)	

Significant p values are in bold.

*PT* peritumoral, *EP* extraperitumoral.

**Figure 1:**
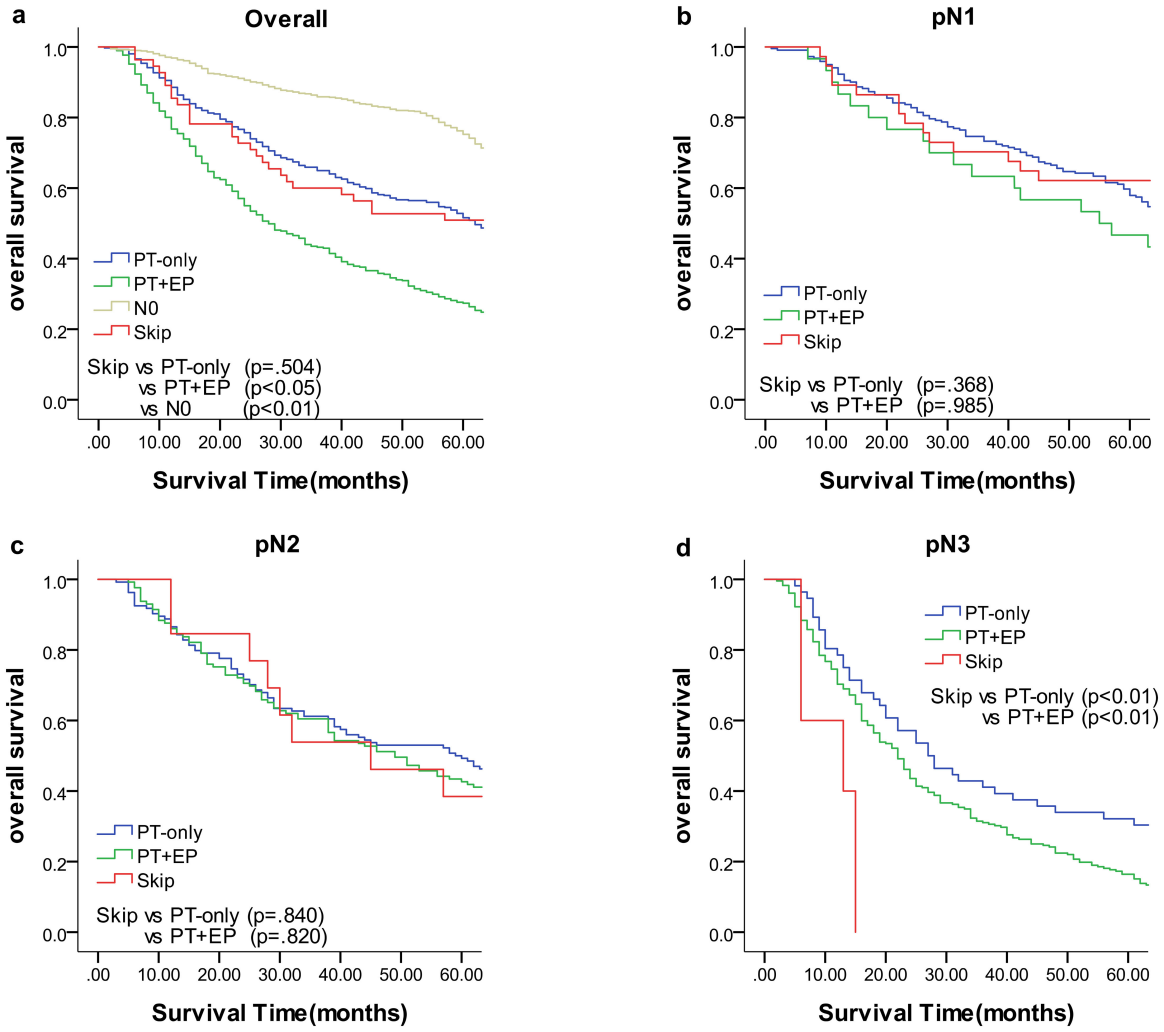
Overall survival of each status of lymph node metastasis with Kaplan–Meier curves: **(a)** for overall stage, **(b)** for N1 stage, **(c)** for N2 stage, and **(d)** for N3 stage. *EP*, extraperitumoral; *PT*, peritumoral.

**Figure 2:**
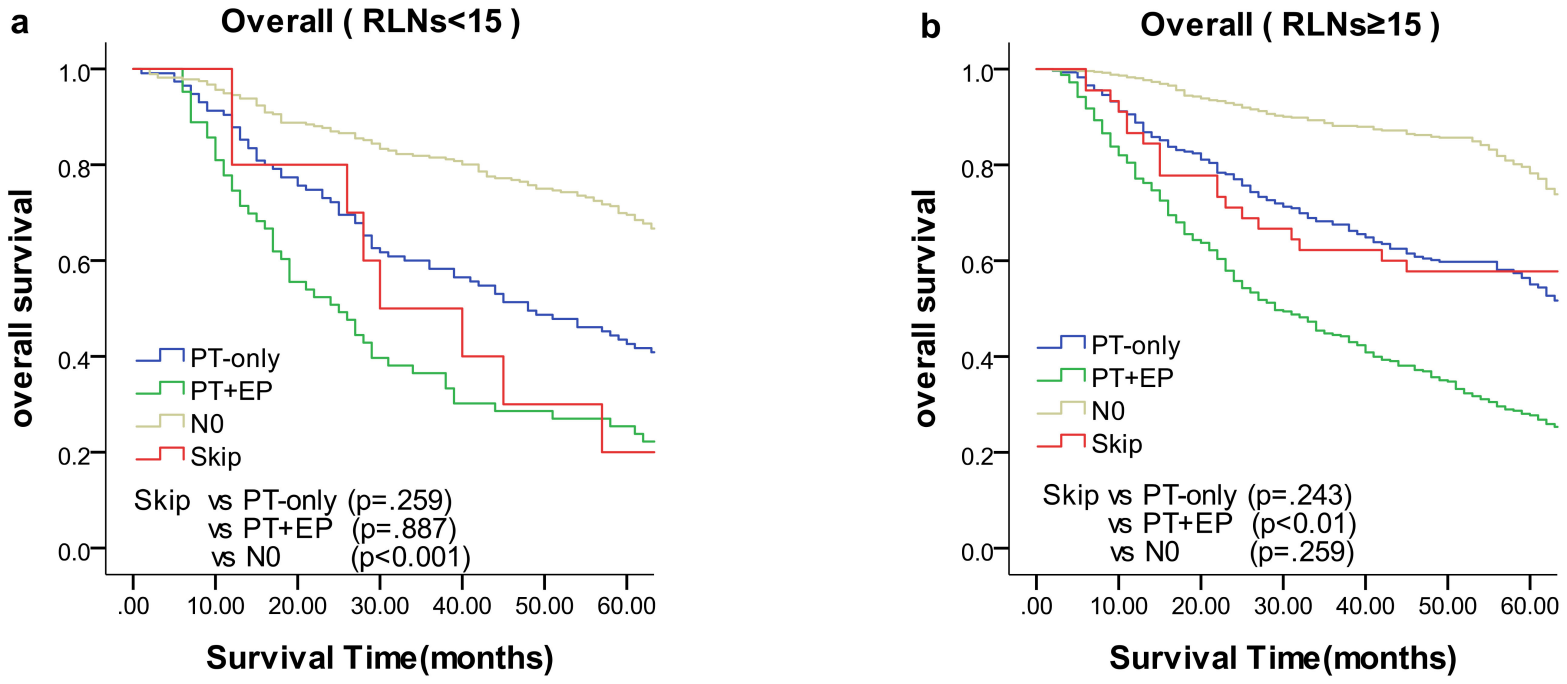
Overall survival of each status of lymph node metastasis with Kaplan–Meier curves: **(a)** for overall stage with RLNs <15, **(b)** for overall stage with RLNs ≥15. *EP*, extraperitumoral; *PT*, peritumoral; *RLNs*, retrieved lymph nodes.

**Figure 3:**
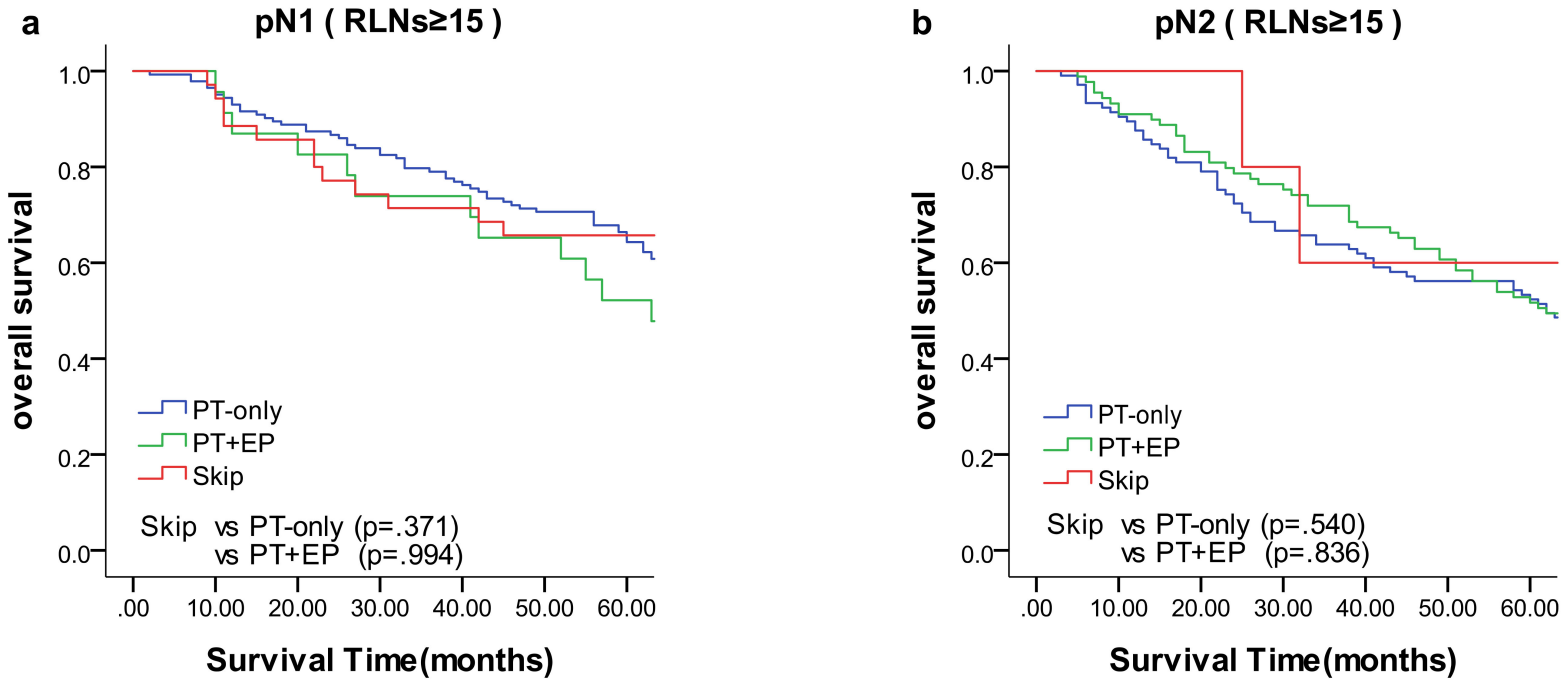
Overall survival of N1 and N2 status of lymph node metastasis with Kaplan–Meier curves: **(a)** for N1 stage with RLNs ≥15, **(b)** for N2 stage with RLNs ≥15. *EP*, extraperitumoral; *PT*, peritumoral; *RLNs*, retrieved lymph nodes.

We compared the results of the 5-year DFS in the 4 groups with respect to overall stage, in 3 groups (skip metastasis, PT-only, and PT+EP groups) with respect to pN1, pN2, and pN3 stages, respectively (**Figure 1**). The prognosis of the skip metastasis group was similar to the PT-only group, but better than the PT+EP group with respect to overall stage. The prognosis of patients in the skip metastasis group was similar to patients in the PG-only and PG+EP groups with respect to pN1 and pN2 stages, and worse than patients in the PG-only and PG+EP groups with respect to pN3 stage. We further analyzed the effect of RLNs on the prognosis of gastric cancer patients, and the results are shown in **Figures 2**, **3**. We identified the point at which the survival times of the skip metastasis or PT-only groups were longer than the PT+EP group with respect to overall stage with the number of RLNs > 15; however, we were not able to distinguish the survival time difference between the skip metastasis and PT-only groups or the skip metastasis and PT+EP groups when the number of RLNs was < 15 with respect to overall, pN1, and pN2 stages.

**Figure 4:**
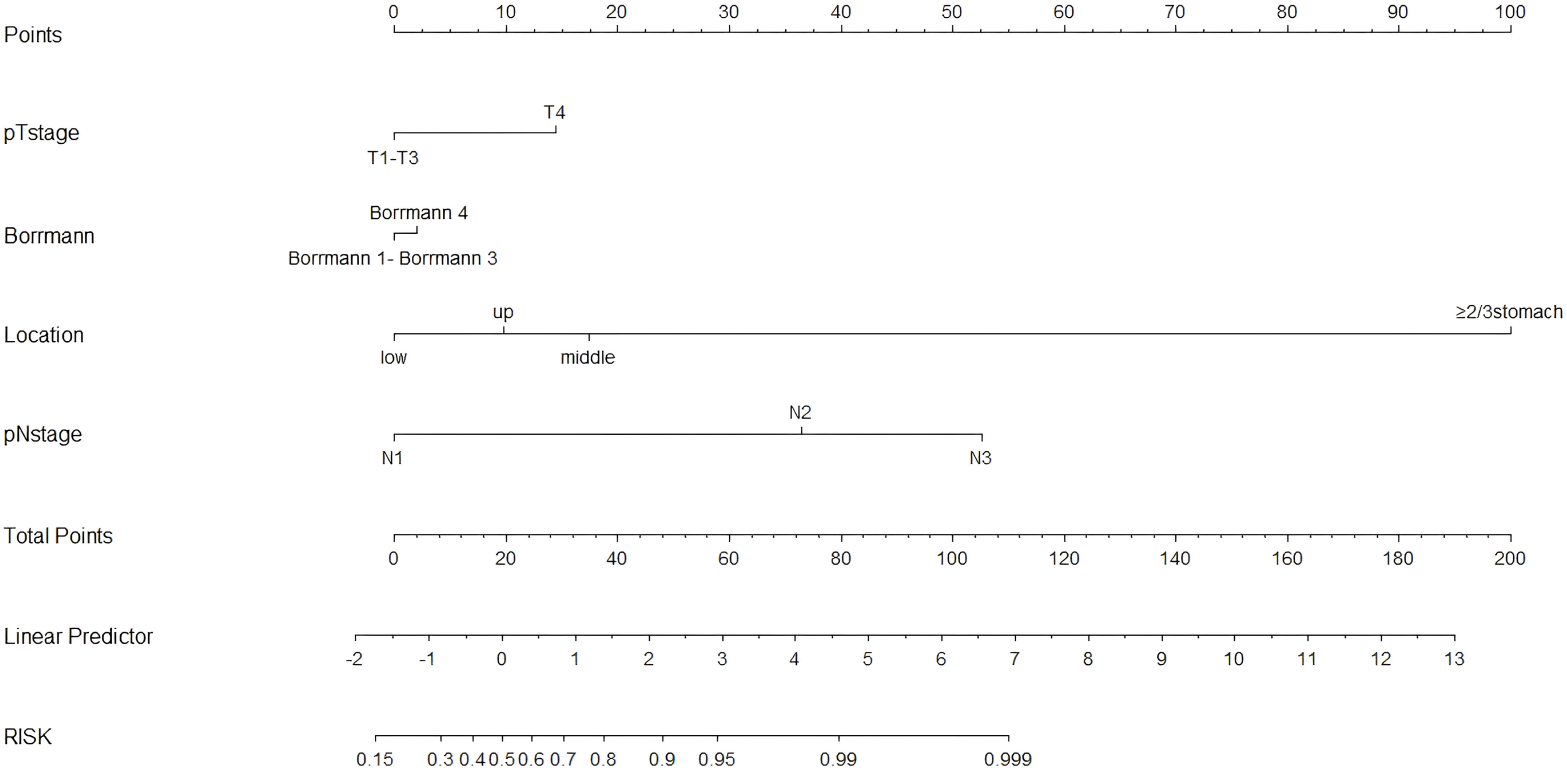
Nomogram for predicting skip lymph node metastasis in gastric cancer: skip group *versus* PT+EP group. The total score for each patient is calculated by drawing a vertical line from the appropriate point for each predictor down to the score scale, and summing these scores. A vertical line is drawn from the total score scale to the predicted probability scale of skip metastasis.

**Figure 5:**
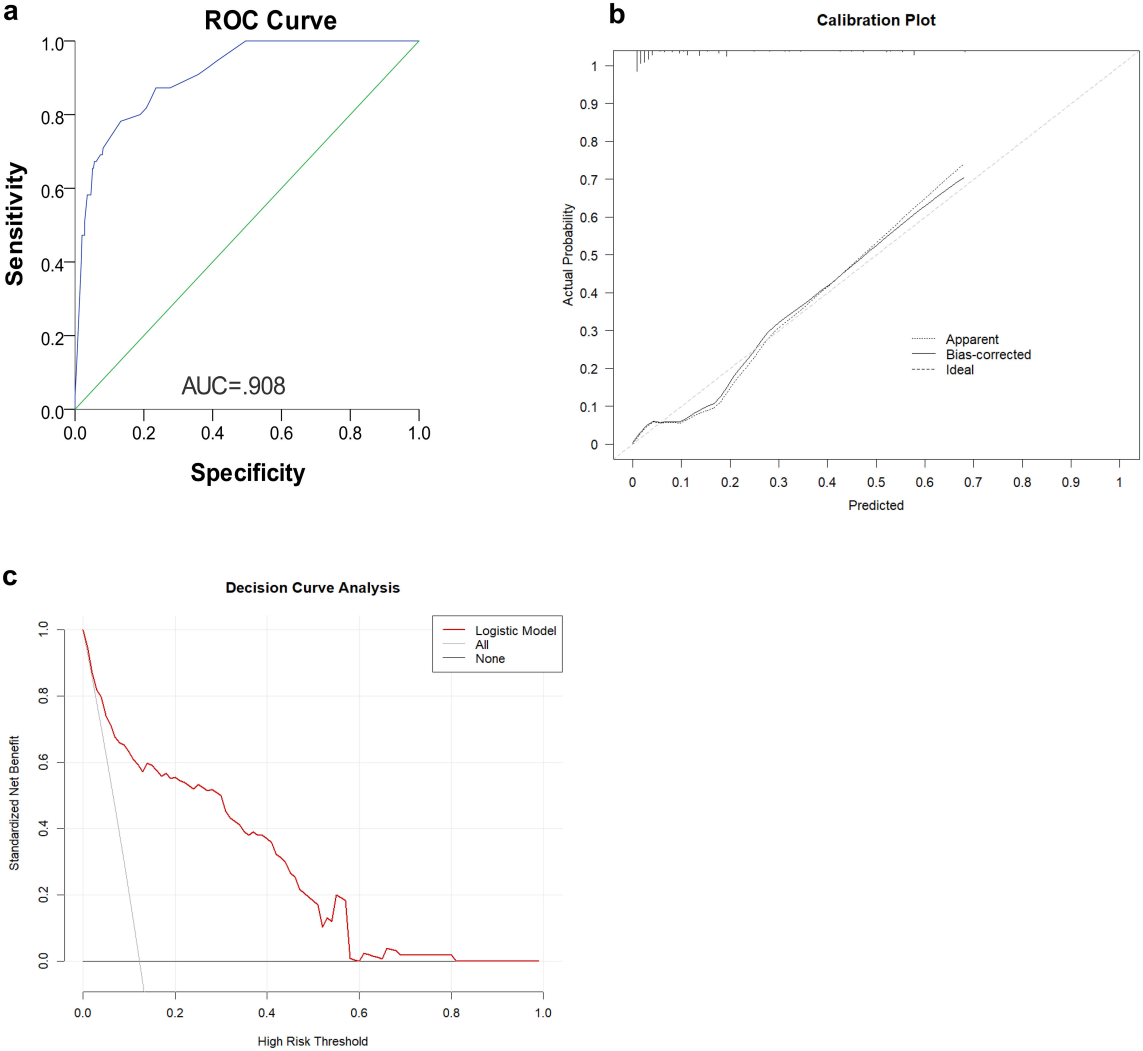
**(a)** Receiver operating characteristic (ROC) curves representing the discriminatory ability of the nomogram, revealed a good concordance to estimate skip metastasis. **(b)** Calibration curve of the skip metastasis nomogram The plot compares predicted probabilities (x-axis) against actual observed frequencies (y-axis). **(c)** Decision curve analysis (DCA) for clinical utility assessment The y-axis shows standardized net benefit, calculated as the ratio of true positives to false positives weighted by threshold probability.

The nomogram (**Figure 4**) was improved when constructed based on the skip group versus PT+EP group and provided a good range of skip metastasis risk to distinguish the prognosis and treatment of the skip metastasis versus PT+EP groups. The ROC curve was used to confirm the ability of the nomogram to predict the LN status of the skip metastasis versus PT+EP groups (**Figure 5a**), and the AUC was 0.908. We further calculated the calibration curve and Decision Curve Analysis, demonstrating the accuracy of the predictive model and the favorable practical impact of the prediction results on clinical decision-making (**Figures 5b, c**).

## 4 Discussion

The incidence of skip metastasis was 6.4% among the patients with metastatic LNs, and 3.3% among the overall gastric cancer population, which was consistent with previous reports; specifically, skip metastasis occurs in 1.8%-6.7% of gastric cancer patients (15, 18, 20, 21). The most common skip metastasis LN groups were the No. 7 (50.9%), No. 8a (32.7%), No. 9 (21.8%), and No.1 groups (20.0%), as shown in **Table 2**. In previous reports, the most frequent skip metastasis LN groups were concentrated in Nos.1, 3, 7, 8a, and 9 groups (22–24).

Regarding the skip metastasis versus N0 group, there are independent factors (pT stage, Lauren type, and histologic findings); however, the N0 group has a tendency to an earlier pT stage (T1, 43.0%, T4, 12.5%) which was in contrast to the other three groups, and was also a different trend with respect to the Lauren type for the N0 group and other three groups (**Table 1**). Due to the different trend of pT stage and Lauren type between the skip metastasis and N0 group, the nomogram did not display a comprehensive range of skip metastasis risk. We discussed that the nomogram of the skip metastasis versus the PT+EP group has practical application and operability (**Figure 4**). The statistical model was used to predict the risk of skip metastasis. Predictors of skip metastasis status identified by multivariable logistic regression and chi-square analyses were pN stage, Borrmann type, pT stage, and location in the stomach. ROC curve analysis of the nomogram revealed good concordance and reliability to estimate the status of skip metastasis (**Figure 5**). The most common skip metastasis groups were Nos. 7 (50.9%), 8a (32.7%), and 9 (21.8%), which is consistent with previous reports (11, 21, 25). We should pay more attention to these LN metastasis groups when skip metastasis is at high risk. In contrast, when the risk is low, which suggests a high risk for metastatic LNs localized in stations I and II, and a worse prognosis.

Dihge et al. (26) applied molecular subtype, patient age, mode of detection, tumor size, presence of multifocality, and vascular invasion for the preoperative prediction of axillary nodal status in patients with breast cancer. Zhao et al. (27) used tumor size, differentiation, ulcerations, combined tumor makers, lymphovascular invasion, and depth of invasion as predictors of LN metastasis in early gastric cancer patients. Zheng et al. (10) used age, macroscopic type, size, histologic findings, differentiation, ulcerations, lymphovascular invasion, and depth of invasion for predicting LN metastasis in early gastric patients. Indeed, predictors of LN metastasis for different tumor types vary widely. Even for different stages of the same tumor type, the predictors are not always the same. No example of prediction model for LN skip metastasis in gastric cancer has been reported as a reference. According to the area under ROC curve of the prediction model (AUC = 0.908), the combination of predictors was reasonable.

We concluded that the location of LN metastasis had an impact on the treatment and prognosis of gastric cancer patients. However, LN metastasis staging (pN staging) based on the number of LN metastases is accepted by clinicians and gastric cancer researchers worldwide, included in TNM staging of the 8th edition of the UICC/AJCC and the 15th edition of JGCA staging (28, 29).

Given the global gastric cancer database updates and improvements in the treatment of gastric cancer, pN staging is continually improved at the same time (30, 31). Since 1968, UICC/AJCC has promulgated eight versions of TNM staging system for gastric cancer, with pN staging in editions 1-4 was based on the distance between LN diffusion and primary tumor (3 cm as the boundary). Since the 5th edition of UICC/AJCC, the change in pN staging was based on optimization of the number of metastatic LNs in each substage (32). In 2016, the current 8th edition of UICC/AJCC for pN staging underwent adjustments. Specifically, pN staging was changed into a comprehensive staging system that includes clinical staging (cTNM), pathologic staging (pTNM), and pathologic staging after neoadjuvant therapy (ypTNM), and the number of RLNs was adjusted from ≥ 15 to ≥ 30 (33–35). The controversy about pN staging of gastric cancer has never stopped, and relevant retrospective studies have been carried out on optimization of UICC/AJCC staging and the significance of JGCA staging (31, 36).

Numerous studies have shown that the number of RLNs can affect the stability of pN staging in clinical application (37–40). It has been proposed that the metastatic LN ratio (rN) reduces stage deviation (41). LN metastasis rate (rN) staging is based on the ratio of the number of positive LNs-to-the total number of RLNs. Moreover, rN is a new staging system that emerged in the context of accurate evaluation of LN metastasis (42, 43). The present study divided rN staging as follows: rN0, no LN metastasis; rN1, LN metastasis rate < 30%; rN2, LN metastasis rate ≥ 30% and < 60%; and rN3 stage, LN metastasis rate ≥ 60% (44). Researchers are of the opinion that picking up LNs as much as possible ensures the accuracy of pN staging (39). The bias of all pN stages in patients with RLNs <15 is higher than in patients with sufficient RLNs; however, rN staging reduces pN staging bias (45–47). Many researchers support rN staging combined with the TNM staging system, which can improve the limitations of pN staging when the number of RLNs is insufficient. According to previous reports, the clinical application of pN staging is simple and intuitive, and consistent with TNM staging of other cancer types (45, 47). rN staging is more accurate for prognosis (48, 49). Because the majority of the previous studies enrolled the sample population from a single center or a Western population, the threshold for rN staging varies (38, 50). The appropriate threshold for staging warrants additional multi-center studies. The different results among these centers also reflect the limitations of staging only based on the number of LN metastases.

The present study demonstrated that the prognosis of skip metastasis for an RLN ≥15 with respect to the overall stage was similar to the PT-only group, but was better than the PT+EP group and worse than the N0 group, which is in agreement with previous studies (18, 51). In the case of a certain range of RLN, the location of LN metastasis affected the prognosis of gastric cancer. The prognosis of patients in the skip metastasis group was not statistically different compared with patients in the PG-only and PG+EP groups with respect to rN1 and rN2 stages (**Figure 3**).

In the past, the clinical characteristics and location of LN metastasis, such as solitary LN metastasis of gastric cancer patients, have been widely investigated to improve the surgical outcomes and follow-up care (52–54); however, there was no predictive model of skip metastasis. For the first time, we established a nomogram for measuring the risk of skip metastasis. We provided a model to identify the factors and results of skip metastasis risk to facilitate optimal LN dissection and individualization of gastric cancer treatment (18, 55). The location of LN metastasis should be considered to improve the current staging, particularly as contemporary guidelines move toward personalized risk stratification (56).

Some limitations of our study should be emphasized here. It is possible that the database we used was influenced by confounding factors due to the retrospective nature of this study. Moreover, there might be some selection bias caused by the single-center cohort. Finally, innovation can be associated with imperfection due to a lack reference. We are looking forward to a more reliable prediction model of LNs status to assist individual treatment of patients with gastric cancer (56).

## Data Availability

The datasets presented in this article are not readily available because the data that support the findings of this study are available from the corresponding author with the permission of the First Affiliated Hospital of China Medical University. Requests to access the datasets should be directed to Zhi Zhu, zhuzhi@cmu.edu.cn.
